# The effect of anti-angiogenic agents on overall survival in metastatic oesophago-gastric cancer: A systematic review and meta-analysis

**DOI:** 10.1371/journal.pone.0172307

**Published:** 2017-02-21

**Authors:** David L. Chan, Katrin M. Sjoquist, David Goldstein, Timothy J. Price, Andrew J. Martin, Yung-Jue Bang, Yoon-Koo Kang, Nick Pavlakis

**Affiliations:** 1 Department of Medical Oncology, Royal North Shore Hospital, Sydney, New South Wales, Australia; 2 National Health and Medical Research Council Clinical Trials Centre, University of Sydney, Sydney, New South Wales, Australia; 3 Department of Medical Oncology, Cancer Care Centre, St George Hospital, Sydney, New South Wales, Australia; 4 Department of Medical Oncology, Prince of Wales Hospital, Sydney, New South Wales, Australia; 5 Department of Medical Oncology, The Queen Elizabeth Hospital and University of Adelaide, Australia; 6 Department of Internal Medicine, Seoul National University College of Medicine, Seoul, South Korea; 7 Department of Oncology, Asan Medical Centre, University of Ulsan College of Medicine, Seoul, South Korea; National Cancer Center, JAPAN

## Abstract

**Background:**

Studies of anti-angiogenic agents (AAs), combined with chemotherapy (chemo) or as monotherapy in metastatic oesophago-gastric cancer (mOGC), have reported mixed outcomes. We undertook systematic review and meta-analysis to determine their overall benefits and harms.

**Methods:**

Randomized controlled trials in mOGC were sought investigating the addition of AAs to standard therapy (best supportive care or chemo). The primary endpoint was overall survival (OS) with secondary endpoints progression-free survival (PFS), overall response rate (ORR) and toxicity. Estimates of treatment effect from individual trials were combined using standard techniques. Subgroup analyses were performed by line of therapy, region, age, performance status, histological type, number of metastatic sites, primary site, mechanism of action and *HER2* status.

**Results:**

Fifteen trials evaluating 3502 patients were included in quantitative analysis. The addition of AAs was associated with improved OS: HR 0·81 (95% CI 0·75–0·88, p<0·00001) and improved PFS: HR 0·68 (95% CI 0·63–0·74, p<0·00001). Subgroup analyses favoured greater benefit for OS in 2^nd^/3^rd^ line settings (HR 0·74) compared to 1^st^-line settings (HR 0·91) (X^2^ = 6·00, p = 0·01). OS benefit was seen across all regions—Asia (HR 0·83) and rest of world (HR 0·75)—without significant subgroup interaction. Results from 8 trials evaluating 2602 patients were pooled for toxicity > = Grade 3: with OR 1·39 (95% CI 1·17–1·65).

**Conclusions:**

The addition of AAs to standard therapy in mOGC improves OS. Improved efficacy was only observed in 2^nd^- or 3^rd^-line setting and not in 1^st^-line setting. Consistent OS benefit was present across all geographical regions. This benefit is at the expense of increased overall toxicity.

## Introduction

Gastric cancer is the fifth most common malignancy diagnosed worldwide and the 3^rd^ leading cause of cancer mortality[1]. Median survival of patients with metastatic oesophago-gastric cancer (mOGC) is less than 12 months with currently available treatment[2]. The survival of patients with HER-2 positive mOGC is better with a median survival of 16 months[3]. Anti-angiogenic agents (AAs), both antibodies and tyrosine kinase inhibitors (TKIs), targeting angiogenic signalling pathways such as *VEGF* or its receptors, have been tested in the treatment of mOGC. The initial first line phase III trial with bevacizumab [4] failed to show survival benefit, but recently reported phase III trials with ramucirumab [5] and apatinib [6] have shown improved overall survival (all three trials showed benefit for progression-free survival). We undertook this systematic review and meta-analysis to evaluate the overall effect of anti-angiogenic agents, in combination with chemotherapy and as monotherapy, in the treatment of metastatic oesophago-gastric cancer (mOGC), with respect to the outcomes of overall survival, progression-free survival, response rate, toxicity measures and quality of life.

## Methods

We sought to identify randomised controlled trials comparing survival in metastatic oesophago-gastric cancer with the use of anti-angiogenic therapy and chemotherapy versus chemotherapy alone, or the use of anti-angiogenic therapy as monotherapy with best supportive care. To be eligible for consideration, trials needed to restrict enrolment to patients with adenocarcinoma with primary site located in the stomach or gastro-oseophageal junction. Trials were included in the meta-analysis provided they reported at least one of overall survival (OS), progression-free survival (PFS) or overall response rate (ORR), and conducted intention-to-treat analyses. Where specified, *HER2* status of patients enrolled in each trial and subsequent subgroup analyses were noted, but inclusion or exclusion of these patients were not grounds for study exclusion.

We searched PUBMED (1946 to current), EMBASE (1974 to 2014), and the Cochrane Central Register of Controlled trials on December 7 2014 using the following search terms: “esophageal neoplasms”, “stomach neoplasms”, “antineoplastic agents”, “chemotherapy”, “vascular endothelial growth factor”, “VEGF” and specific names of anti-angiogenic agents in clinical use (S1 Methods). We also manually searched for abstracts from major conferences from 2012–2014. The search was completed on December 14 2014. Records were screened by two authors (DC, NP) with unclear abstracts clarified by reading of the full-text article. Articles for which neither the abstract nor full text were available in English were excluded. Authors were not contacted for additional information. This review was not centrally registered. The systematic review and meta-analysis complied with PRISMA guidelines (S2 Methods). A repeat search was performed on September 6 2016 using the same methodology to identify articles published after the initial search.

The primary outcome of interest in this meta-analysis was OS. Secondary outcomes included PFS, ORR, disease control rate (DCR), toxicity, and quality of life (QoL) data where available. Trials were assessed for quality according to the Cochrane risk of bias tool [7].

### Statistical analysis

Two authors (DC, NP) extracted relevant data for each trial into a piloted spreadsheet with the hazard ratio (HR) being extracted as the summary measure of effect for OS and PFS, and event rates for ORR and toxicity. Where multiple subgroups were reported without a summary statistic, these were combined into a single summary statistic using random effects modeling.

A summary measure of treatment effect for each endpoint was obtained by pooling estimates from individual studies with the fixed-effects model and inverse-variance weighting. The applicability of the standard fixed-effects model, as well as evidence of heterogeneity of effect across subgroups, was evaluated using the Q-test and the I^2^ statistic. Funnel plots were prepared to help identify signs of publication bias. If the hazard ratio was not available in the published text but Kaplan-Meier curves were, the hazard ratio was derived using the method outlined by Parmar et al [8]. The random effects model was used for sensitivity analysis if substantial statistical or clinical heterogeneity was identified. Data were analysed with software provided by the Cochrane Library (Rev Man 5.3, downloaded March 2015).

All eligible studies reporting hazard ratio for OS, PFS or ORR were included for the primary analysis, and those considered at high risk of bias were excluded in a secondary sensitivity analysis. We also included studies in pre-planned subgroup analyses by line of therapy (1^st^ vs 2^nd^-3^rd^), geographic region (Asia vs rest of world), Age (<65 vs > = 65), histological subtype (intestinal, diffuse or other), performance status (0 vs > = 1), number of metastatic sites (< = 2 vs >2), primary site (gastric, gastro-oesophageal junction or oesophageal), mechanism of action (targeting *VEGF*, targeting *VEGFR* and/or other receptors, and other) and *HER2* status (positive or negative).

## Results

### Study selection

**Fig 1:**
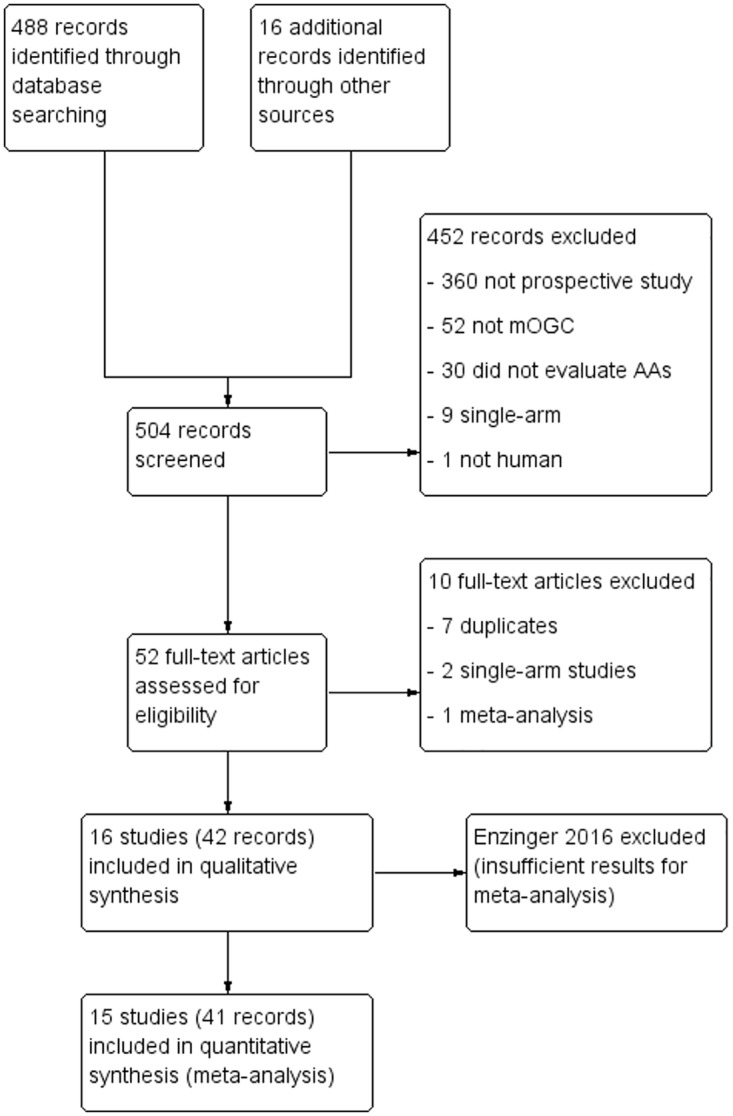
Study flow diagram.

The initial search strategy yielded 503 entries (Fig 1). 51 articles were retrieved in full text, and consequently 15 studies were identified [4–6,9–20]. (Table 1).

**Table 1:** Summary of included studies.

Name	Author	Line	Experimental arm	Control arm	Number of patients	OS HR (95% CI)	PFS HR (95% CI)
AVAGAST[4]	Ohtsu(2011)	1^st^	CX + bevacizumab	CX	774 (387 CX+B, 387 CX)	0·87 (0·73–1·03)	0·80 (0·68–0·93)
Eatock 2013 [[10]	Eatock (2013)	1^st^	CX + trebananib 10mg/kg, CX + trebananib 3mg/kg	CX	171 (56 10mg/kg, 59 3mg/kg, 56 CX alone)	N/A	0·98 (0·67–1·43)
Koizumi 2013 [13]	Koizumi (2013)	1^st^	S1/Cisplatin + orantinib	S1/Cisplatin	91 (45 S1/Cisplatin + orantinib, 46 S1/Cisplatin)	0·74 (0·46–1·19)	1·23 (0·74–2·05)
AVATAR [16]	Shen (2014)	1^st^	CX+ Bevacizumab	CX	202 (100 CX+B, 102 CX)	1·11 (0·79–1·56)	0·89 (0·66–1·21)
STARGATE [19]	Kang (2014)	1^st^	CX+sorafenib	CX	195 (97 CX+S, 98 CX)	0·93 (0·65–1·31)	0·92 (0·67–1·27)
Yoon 2014 [18]	Yoon (2014)	1^st^	FOLFOX+ ramucirumab	FOLFOX	168 (84 FOLFOX+R, 84 FOLFOX)	1·08 (0·73–1·58)	0·98 (0·69–1·37)
PaFLO [20]	Thuss-Patience (2015)	1^st^	5-Fluorouracil + Oxaliplatin + Pazopanib	5-Fluorouracil + Oxaliplatin	78 (51 5-FU + O + P, 27 5-FU + O)	0·80 (0·44–1·48)	0·93 (0·56–1·54)
Jiang 2009 [12]	Jiang (2009)	1^st^	XELOX+ endostatin	XELOX	42 (20 XELOX+E, 22 XELOX)	N/A	N/A
RAINBOW[5]	Wilke (2012)	2^nd^	Paclitaxel+ ramucirumab	Paclitaxel	665 (330 P+R, 335 P)	0·81 (0·68–0·96)	0·63 (0·54–0·75)
Yi 2012 [17]	Yi (2012)	2^nd^	Docetaxel+ sunitinib	Docetaxel	107 (56 D+S, 49 D)	0·94 (0·60–1·49)	0·77 (0·52–1·16)
REGARD [11]	Fuchs (2014)	2^nd^	Ramucirumab	Placebo	355 (238 Ram, 117 Placebo )	0·78 (0·60–1·00)	0·48 (0·38–0·62)
AIO Moehler 2013 [14]	Moehler (2013)	2^nd^-3^rd^	FOLFIRI+ Sunitinib	FOLFIRI	90 (45 FOLFIRI+S, 45 FOLFIRI)	0·82 (0·50–1·34)	1·11 (0·70–1·74)
INTEGRATE[15]	Pavlakis (2015)	2^nd^-3^rd^	Regorafenib	Placebo	147 (97 Reg, 50 Placebo)	0·74 (0·51–1·08)	0·40 (0·28–0·59)
Li 2013 [9]	Li (2013)	3^rd^	Apatinib	Placebo	144 (48 Apatinib 850, 48 Apatinib 425BD, 48 PBO)	425mg bd: 0·41 (0·24–0·72). 850mg od: 0·37 (0·22–0·62)	425mg bd: 0·21 (0·11–0·38). 850mg od: 0·18 (0·10–0·34)
Qin 2014 [6]	Qin (2014)	3^rd^	Apatinib	Placebo	273 (182 apatinib, 91 PBO)	0·71 (0·54–0·94)	0·44 (0·33–0·60)

Abbreviations: CX—Cisplatin + capecitabine, B—Bevacizumab, FOLFOX– 5-fluorouracil/folinic acid + oxaliplatin, FOLFIRI—fluorouracil/folinic acid + irinotecan, XELOX—capecitabine+oxaliplatin, Ram—ramucirumab, 5-FU + O– 5-Fluorouracil + Oxaliplatin, P—Pazopanib

The repeat search identified three full publications for trials previously reported in abstract form only (INTEGRATE, Qin 2014, Moehler 2013)[6,14,15]. A previously identified study (PaFLO) reported results in abstract form, which were then incorporated into the meta-analysis[20]. A new study (Enzinger 2016) was identified, but the required information for analysis could not be extracted from the abstract, leaving the same 15 studies for quantitative analysis[21].

### Risk of bias

#### Overall risk of bias

The risk of bias for three trials (ref Kang, Yoon, Thuss-Patience) [18–20]) (21) could not be fully established due to insufficient information as they have only been published in abstract form to date. Nine trials (Eatock, REGARD, Li 2013, AVAGAST, AVATAR, RAINBOW, INTEGRATE, Qin 2013, Moehler 2013)[4–6,9–11,14–16] were rated at low risk of bias. Three trials were rated at high risk of bias (Koizumi 2013, Yi 2012 and Jiang 2009) as they were all open-label studies which employed unblinded assessment of progression as the primary endpoint[12,13,17]. (S1 Fig)

#### Funnel plots

There was no definite evidence of publication bias based on the shape of the funnel plot produced using estimates of effect on OS from the 14 studies reporting this statistic. (S2 Fig)

### Overall results—AAs improve OS, PFS, ORR and DCR but also increase rates of toxicity

**Fig 2:**
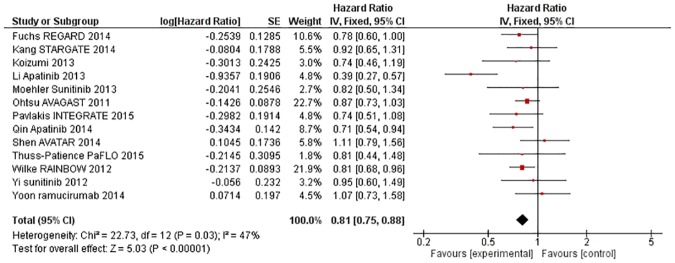
Forest plot—Overall Survival—all trials.

Thirteen studies were included in the analysis for overall survival (n = 3289). The pooled HR was 0·81 (95% CI 0·75–0·88, p<0·00001, Fig 2). Eatock 2013 and Jiang 2009 did not report overall survival in sufficient detail to allow inclusion in the meta-analysis. There was evidence of moderate heterogeneity (X^2^ = 22·73, p = 0·03, I^2^ = 47%); however, a random effects model yielded a comparable pooled estimate (HR = 0.80, 95% CI 0·71–0·91, p = 0·0005) to that of the fixed effects model.

**Fig 3:**
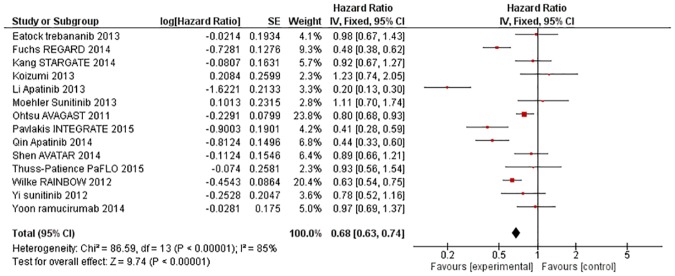
Forest plot—Progression-free Survival—all trials.

Pooled analysis of PFS performed on data from 14 RCTs comprising 3459 patients yielded a significant HR of 0·68 (95% CI 0·63–0·74, p<0·00001, Fig 3). (Jiang 2009 again did not report PFS in sufficient detail to include in the meta-analysis). There was statistical evidence of substantial heterogeneity (X^2^ = 86·59, I^2^ = 85%, p<0·00001), however a random effects model yielded a comparable pooled estimate (HR = 0·69, 95% CI 0·56–0·85, p = 0·0006).

Analysis of 13 RCTs (3086 patients) showed improved response rate with the addition of AAs with OR of 1·44 (95% CI 1·20–1·71, p<0·0001). The pooled response rate was 27.5% (476/1730) in the experimental arm and 25.9% (351/1356) in the control arm. There was no evidence of significant heterogeneity (X^2^ = 16.04, I^2^ = 25%. P = 0·19).

DCR (a composite of confirmed RECIST SD/PR/CR) was also improved with OR of 2·01 (95% CI 1·70–2·37, p<0·00001). The pooled DCR was 64.5% (1116/1730) in the experimental arm and 56.9% (772/1356) in the control arm. There was evidence of substantial heterogeneity (X^2^ = 34·05, I^2^ = 65%, p = 0·0007), however a random effects analysis yielded a comparable pooled estimate (OR 2·00, 95% CI 1·47–2·73, p<0·0001).

Overall Grade 3–5 toxicity (8 RCTs, 2602 patients) was increased with addition of AAs, with a statistically significant OR of 1·39 (95% CI 1·17–1·65, p = 0·0002). The pooled toxicity rate was 70.4% (1013/1438) in the experimental arm and 65.2% (759/1164) in the control arm. There was evidence of substantial heterogeneity (X^2^ = 29·58, I^2^ = 76%, p<0·0001), and random effects analysis yielded a comparable pooled estimate but this was no longer statistically significant (OR 1·39, 95% CI 0·94–2·04, p = 0·10, S3 Fig). Therefore, the finding of increased toxicity with AAs is less statistically certain than the findings of increased efficacy noted above. This may in part be due to the pooling of toxicity of different agents with different toxicity profiles.

Common adverse events pertinent to anti-angiogenic agents include hypertension, hand-foot syndrome and thrombosis, depending on the class of agent (mAb vs TKI), and are described as follows: The majority of studies investigating MAb reported an increase in Grade 3+ hypertension (for example, 6% vs <1% in AVAGAST and 14% vs 2% in RAINBOW), but no definite increase in GI perforation (2% vs <1% in AVAGAST, 1% vs 0% in AVATAR, 1% vs <1% in RAINBOW). The majority of studies investigating TKIs reported an increase in Grade 3 hypertension (10% vs 2% in INTEGRATE, 5% vs 0% in Qin 2014), Grade 3+ hand-foot syndrome (9% vs 0% in Qin 2014, 9% vs 0% in Li 2013), and Grade 3+ diarrhoea (11% vs 4% in Koizumi 2013, 11% vs 2% in Yi 2012). Thrombosis (both venous and arterial) and bleeding were not significantly increased in the AA arm across all studies, regardless of mechanism of action.

### Subgroup analyses

#### AAs improve OS in second line settings and beyond, but not first line settings

**Fig 4:**
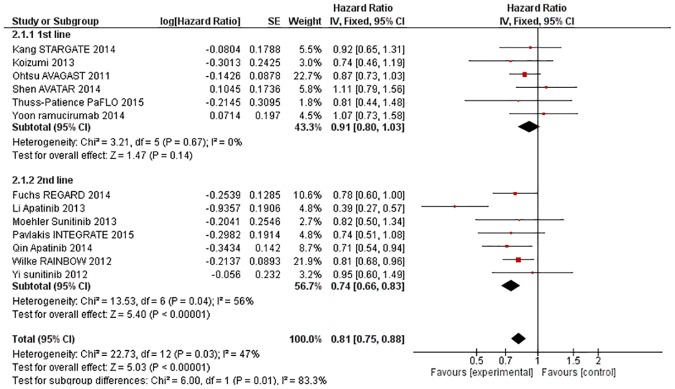
Forest plot—Overall Survival—by line of therapy.

The effect of AA on OS was greater in the second-line setting than in the first-line setting (X^2^ = 6·00, p = 0·01, Fig 4). The estimated HR was 0·91 (95% CI 0·80–1·03, p = 0·14) in the first-line setting, and 0·74 (95% CI 0·66–0·83, p<0.00001) in the second-line setting. There was some evidence of heterogeneity overall (X^2^ = 22·73, p = 0·03, I^2^ = 47%) and also amongst the trials performed in the second-line setting (X^2^ = 13·53, p = 0·04, I^2^ = 56%), however AA still provided greater OS benefit in the second-line setting on random effects modelling (HR 0·72, 95% CI 0·60–0·86, p = 0.0003) and the interaction remained significant (X^2^ = 4·42, p = 0·04).

The effect of AA on PFS was greater in the second-line setting than in the first-line setting (X^2^ = 39·33, p<0·00001, S4 Fig). The estimated HR was 0·88 (95% CI 0·79–0·98, p = 0·02) in the first-line setting, and 0·54 (95% CI 0·48–0·60, p<0·00001) in the second-line setting. There was evidence of substantial heterogeneity overall (X^2^ = 86·59, p<0·00001, I^2^ = 85%) and amongst the trials performed in the second-line setting (X^2^ = 43·22, p<0·00001, I^2^ = 86%), but the above interaction remained significant on random effects modelling (X^2^ = 10·14, p = 0·001), and PFS benefit in the second-line was preserved (HR 0·51, 95% CI 0·38–0·70, p<0.0001).

The effect of AA on ORR was greater in the second-line setting than in the first-line setting (X^2^ = 5·13, p = 0·02). The estimated OR was 1·24 (95% CI 1·00–1·54, p = 0·05) in the first-line setting, and 1·92 (95% CI 1·41–2·62, p<0·0001) in the second-line setting. There was evidence of moderate heterogeneity (X^2^ = 9·68, p = 0·14, I^2^ = 38%) amongst the trials performed in the second-line setting, but the estimate of ORR benefit remained statistically significant on random effects modelling (OR 1·84, 95% CI 1·04–3·25, p = 0·03).

The effect of AA on DCR was greater in the second-line setting than in the first-line setting (X^2^ = 18·91, p<0·0001). The estimated OR for response was 1·36 (95% CI 1·07–1·73, p = 0·01) in the first-line setting, and 2·88 (95% CI 2·27–3·66, p<0·00001) in the second-line setting. Heterogeneity was noted overall (X^2^ = 34·05, p = 0·0007, I^2^ = 65%) as well as amongst second-line trials (X^2^ = 9·72, p = 0·14, I^2^ = 38%), but random effects modelling showed preservation of DCR benefit in second-line trials (OR 2·90, 95% CI 2·08–4·04, p<0.0001) and a persistent subgroup interaction statistic (X^2^ = 11·73, p = 0·0006).

The addition of AAs increased Grade 3–5 toxicity more in the second-line setting than in the first-line setting (X^2^ = 13·28, p = 0·0003): The estimated OR was 0·94 (95% CI 0·72–1·24, p = 0·67) in the first-line setting, and 1·82 (95% CI 1·45–2·27, p<0·00001) in the second-line setting. There was evidence of heterogeneity overall (X^2^ = 29·58, p = 0·0001, I^2^ = 76%), as well as amongst first-line (X^2^ = 3·11, p = 0·21, I^2^ = 36%) and second-line (X^2^ = 13·52, p = 0·009, I^2^ = 70%) trials. Random-effects modelling showed that the incidence of toxicity was still not increased in first-line trials (OR 0·96, 95% CI 0·66–1·41, p = 0·84), still increased in second-line trials (OR 1·71, 95% CI 1·08–2·69, p = 0·02), but that the subgroup interaction was no longer statistically significant (X^2^ = 3·56, p = 0·06). Therefore, there may not be a true differential impact on toxicity between the use of AAs in first versus second-line settings. (S5 Fig)

#### AAs improve OS as monotherapy more than in combination with chemotherapy

**Fig 5:**
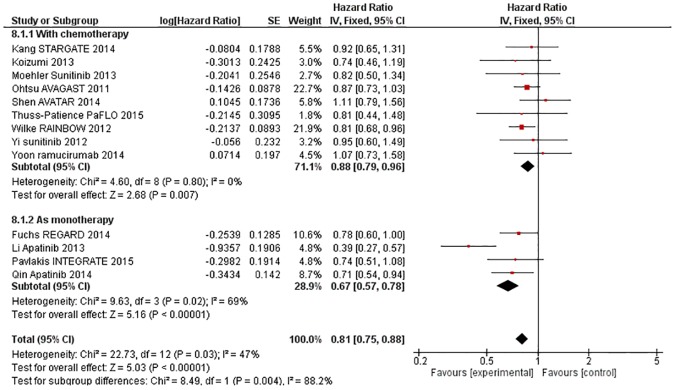
Forest plot—Overall Survival—with chemotherapy vs as monotherapy.

The effect of AA on OS was greater when used as monotherapy (against placebo) than in combination with chemotherapy (against chemotherapy) (X^2^ = 8·49, p = 0·004, Fig 5). The estimated HR was 0·88 (95% CI 0·79–0·96, p = 0·007) in trials of AAs with chemotherapy, and 0·67 (95% CI 0·57–0·78, p<0·00001) in trials of AAs as monotherapy. There was evidence of substantial heterogeneity overall (X^2^ = 22·73, p = 0·03, I^2^ = 47%) and amongst monotherapy trials (X^2^ = 9·63, p = 0·02, I^2^ = 69%), but random effects modelling showed persistence of both OS benefit in monotherapy trials (HR 0·64, 95% CI 0·49–0·85, p = 0.002) and the subgroup interaction (X^2^ = 4·10, p = 0·04, I^2^ = 75.6%).

The effect of AA on PFS was greater when used as monotherapy than in combination with chemotherapy (X^2^ = 57·17, p<0·00001). The estimated HR was 0·81 (95% CI 0·74–0·88, p<0·00001) in combination trials, and 0·40 (95% CI 0·34–0·47, p<0·00001) in the monotherapy trials. There was evidence of substantial heterogeneity overall (X^2^ = 86·59, p<0·00001, I^2^ = 85%), in combination trials (X^2^ = 15·82, p = 0·07, I^2^ = 43%) and also in monotherapy trials (X^2^ = 13·61, p = 0·003, I^2^ = 78%), but random effects modelling showed that PFS benefit persisted in both groups (combination trials HR 0·86, 95% CI 0·75–0·97, p = 0.02; monotherapy trials HR 0·37, 95% CI 0·26–0·53, p<0·00001) and the subgroup interaction remained statistically significant (X^2^ = 19·43, p<0·0001, I^2^ = 94.9%).

The effect of AA on ORR was not significantly different when used as monotherapy compared to use in combination with chemotherapy (X^2^ = 1·31, I^2^ = 23·7%).

The effect of AA on DCR was greater when used as monotherapy than in combination with chemotherapy (X2 = 18·03, p<0·0001). The estimated OR was 1·60 (95% CI 1·32–1·94, p<0·00001) in the combination trials, and 3·80 (95% CI 2·68–5·39, p<0·00001) in monotherapy trials. Heterogeneity was noted overall (X^2^ = 34·05, p = 0·0007, I^2^ = 65%) as well as amongst the combination trials (X^2^ = 14·96, p = 0·06, I^2^ = 47%), but random effects modelling showed persistence of DCR benefit in combination trials (OR 1·60, 95% CI 1·32–1·94, p<0·00001) and significant subgroup interaction (X^2^ = 18·03, p<0·0001, I^2^ = 94.5%).

The effect of AA on Grade 3–5 toxicity was not significantly different when used as monotherapy compared to use in combination with chemotherapy (X^2^ = 0·07, I^2^ = 0%).

#### AAs improve OS regardless of geographical region (Asia vs rest of world)

**Fig 6:**
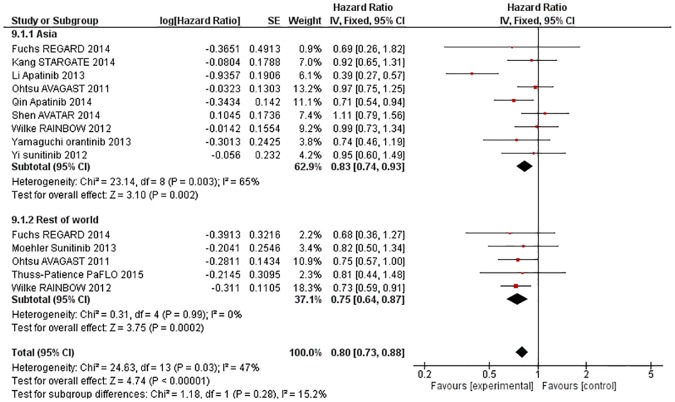
Forest plot—Overall Survival—by region.

Using a fixed-effects model, the effect of AA was not significantly different for patients from Asia compared to patients from the rest of the world for the endpoints of OS (X^2^ = 1·18, p = 0·28, Fig 6) or PFS (X^2^ = 0·28, p = 0·60, S6 Fig).

Comparison of overall response rate by region was not possible given that no trial conducted across regions reported response rate by region and only one trial conducted outside Asia(15) reported response rates.

Comparison of Grade 3–5 toxicity by region was not possible given that all trials reporting toxicity by region were from Asia.

#### AAs improve OS regardless of age

Using a fixed-effects model, the effect of AA was not significantly different for patients with age<65 compared to those with age>65 for the endpoints of OS (X^2^ = 0·01, p = 0·93, S7 Fig), PFS (X^2^ = 1·48, p = 0·22). No studies reported ORR, DCR or Grade 3–5 toxicity by age-defined subgroups.

#### AAs improve OS regardless of performance status

Using a fixed-effects model, the effect of AA was not significantly different for patients with performance status 0 compared to those with performance status> = 1 for the endpoints of OS (X^2^ = 0·62, p = 0·43, S8 Fig) and PFS (X^2^ = 3·53, p = 0·06). No studies reported ORR, DCR or Grade 3–5 toxicity by performance status.

#### AAs improve OS regardless of histological subtype

Using a fixed-effects model, the effect of AA was not significantly different by histological subtype (intestinal, diffuse or other) for the endpoints of OS (X^2^ = 1·04, p = 0·60, S9 Fig) and PFS (X^2^ = 0·51, p = 0·77).

#### AA efficacy was not affected by number of metastatic sites (1–2 vs 3+)

Using a fixed effects model, the effect of AA was not significantly different according to the number of metastatic sites (1–2 versus 3+) for the endpoints of OS (X^2^ = 0·28, p = 0·60, S10 Fig) or PFS (X^2^ = 0·91, p = 0·34).

#### AAs targeting *VEGFR* improve PFS more than ones targeting *VEGF*

There was insufficient data to analyse studies other than those targeting *VEGF* and those targeting *VEGFR* (possibly in addition to other targets). Using a fixed-effects model, the effect of AA was not significantly different for the endpoint of OS (X^2^ = 3·18, p = 0·07) but did favour trials of agents targeting *VEGFR* for the endpoint of PFS (X^2^ = 10·51, p = 0·001, S11 Fig). The estimated PFS HR was 0·82 (95% CI 0·71–0·94, p = 0·004) for *VEGF* agents, and 0·72 (95% CI 0·56–0·68, p<0·00001) for agents targeting *VEGFR*. There was evidence of substantial heterogeneity overall (X^2^ = 83·01, p<0·0001, I^2^ = 86%) and amongst *VEGFR* trials (X^2^ = 72·05, p<0·0001, I^2^ = 86%). Random effects modelling showed persistence of PFS improvement in *VEGFR* trials (HR 0·65, 95% CI 0·49–0·84, p = 0·001) but that the subgroup interaction was no longer statistically significant (X^2^ = 2·27, p = 0·13, S12 Fig). Therefore, this potential interaction should be interpreted with caution.

#### AA efficacy was not affected by the primary site

No studies reported subgroup results for patients with oesophageal carcinoma, and thus meta-analysis was only performed on results for gastric and gastro-oesophageal primary sites. Using a fixed-effects model, the effect of AA on OS was not significantly different by primary site for the endpoints of OS (X^2^ = 0·42, p = 0·52, S13 Fig) or PFS (X^2^ = 3.14, p = 0·08).

#### The effect of *HER2* status on AA efficacy was not able to be determined

None of the identified trials explicitly excluded patients with *HER2* mutations identified on tissue testing, or reported efficacy results by *HER2* status. We were therefore unable to investigate this question.

### Sensitivity analyses

Given the mixed quality of included studies, sensitivity analyses were performed to investigate the impact of excluding studies considered at high risk of bias: Koizumi 2013, Jiang 2009, and Yi 2012 (S1 Fig). Fixed-effects analysis of OS demonstrated a persistently significant HR of 0·81 (95% CI 0·74–0·88, p<0·00001). Exclusion of studies at high or unclear risk of bias and fixed-effects analysis resulted in a similar HR of 0·79 (95% CI 0·72–0·86, p<0·00001). Sensitivity analyses were performed in the above subgroups and results significantly affected are mentioned below. The fixed-effects subgroup analysis by line of therapy for ORR no longer showed significant interaction favouring second-line use (X^2^ = 2·22, p = 0·14). The fixed-effects subgroup analysis by region for ORR no longer showed significant interactions (X^2^ = 3·27, p = 0·07).

We also explored the impact of antiangiogenic type (monoclonal antibody versus tyrosine kinase inhibitor) in a sensitivity analysis. When separated by type, both trials of monoclonal antibodies and trials of tyrosine kinase inhibitors showed preserved, significant benefit in OS and PFS (data not shown).

### Quality of life

Six of the identified trials reported quality of life data (S1 Table) using validated QoL scales (EORTC QLQ-C30 –global and subscales, EORTC QLQ-STO22, Time to deterioration of ECOG> = 2). Significant improvement in quality of life was found with apatinib, in improving insomnia(13) (p = 0·002), ramucirumab in delaying time to deterioration of PS> = 2(10) (p = 0·002) and improving functional functioning and nausea(5) (HR<0·75), bevacizumab in slowing deterioration in pain(4) (p = 0·0068), and endostatin in improving global QoL(11) (p<0·05). Studies reporting no difference in global QoL investigated apatinib(13), ramucirumab(10) and bevacizumab(4). Only one study investigating ramucirumab(5) reported worsening of diarrhoea (HR>1) in the investigational arm.

## Discussion

In terms of the overarching question—whether the use of AAs improves outcomes in mOGC?—our review demonstrates that the use of AAs significantly improves overall survival, progression-free survival, response rate and disease control rate in this disease. The odds of Grade 3–4 toxicity, however, were also significantly increased. Substantial statistical heterogeneity was present in the analyses for the above endpoints, most likely due to the pooling of studies across different lines of therapy investigating AAs with different modes of action (apatinib, ramucirumab, bevacizumab, regorafenib, sunitinib, trebananib, sorafenib, orantinib) and hence different magnitudes of effect. This explanation is strengthened by the observation that heterogeneity was less marked when you examine the pooling trials of agents in the same line of therapy and by the significant subgroup differences found comparing first- and second-line trials.

Pre-defined subgroup analyses demonstrated that the most consistent and statistically certain benefit was found when AAs were used in second-line settings and beyond, and as monotherapy. This benefit, if biologically based, has obvious implications for clinical practice. The superior efficacy of AAs in later line settings might be explained by several factors. Firstly, exposure to and progression after first-line chemotherapy may select and/or promote an angiogenic phenotype—that is, tumour biology may be altered by chemotherapy such that tumours are more sensitive to subsequent targeting of the *VEGF* pathway. Alternatively, patients who fare sufficiently well to enter a second-line trial may have tumour characteristics [22] conferring increased sensitivity to AAs. The use of different agents in first-line versus second-line settings may also be partially contributory. First-line trials investigated bevacizumab, trebananib, ramucirumab, sorafenib and orantinib—a heterogeneous group of agents with diverse targets—whereas trials for second-line settings and beyond generally investigated agents directed against *VEGF* receptor—apatinib, ramucirumab, sunitinib and regorafenib (although the last of these has other targets as well).

Unfortunately, no studies thus far have identified a predictive biomarker to assist patient selection for benefit from AAs. In the AVAGAST first-line study with bevacizumab, high serum *VEGF*-A and low tissue neuropilin-1 were both shown to be prognostic biomarkers, but not necessarily predictive ones[23]. Other studies [11,13,14] have explored other biomarkers (such as *VEGFC*, *VEGFR3*, tissue *VEGFR2*) but these have not been significantly associated with outcome. Detailed biomarker analyses in the INTEGRATE study are ongoing.

Optimization of AAs may depend on a better molecular understanding of gastric adenocarcinoma. Different subtypes of gastric cancer have previously been associated with differential expression of angiogenesis markers e.g. microsatellite high tumours are associated with low rates of angiogenesis marker expression and diffuse type gastric cancer associated with amplification of the fibroblast growth factor receptor 2 gene (*FGFR2*)[24]. *FGFR2* is known to be a potent inducer of angiogenesis[25] and *FGFR2* inhibitors have demonstrated antitumor potential in xenograft models[26,27], although a recently reported phase II study failed to show PFS benefit [28]). Positive *HER2* status was not an exclusion criterion in the studies identified, and given that transtuzumab is efficacious in this population, the above findings are more applicable to the *HER2* negative population. However, we note that less than 20% of patients with mOGC will have overexpression of *HER2*. Increasing understanding of molecular pathways and the dependence of each tumour on angiogenesis may identify subgroups of patients with mOGC who may benefit most from AAs.

The individual analysis of several recent studies of treatment effects by geographic origin have raised the possibility of differential benefit with use of AAs based on region of origin. The first study (AVAGAST) demonstrated a strong geographical difference, with similar suggestions of differential effect in REGARD and RAINBOW in both OS and PFS outcomes. The INTEGRATE trial recently reported a greater PFS benefit with regorafenib in the Korean sub-population compared to Australia/New Zealand/Canada, although benefit was observed in both regions[15]. However, in our meta-analysis, pooling results from all studies, benefit with the use of AAs was seen in OS and PFS regardless of geographic region. This may partly be due to selection bias—for example, more patients in Asia in the INTEGRATE study were treated in the third-line setting, as were patients treated in the apatinib studies. Further research is required to determine whether a true biological difference exists between gastric cancer in patients from different regions that may influence the efficacy of AAs.

No significant subgroup interactions were detected in subgroup analyses of the effect of AAs on overall survival of other known prognostic indicators in mOGC: age, performance status or histological subtype. The primary site did not affect AA efficacy, and whilst AAs targeting *VEGFR* showed possibly greater PFS impact compared to those targeting *VEGF*, this interaction was not significant on random effects modelling and is only based on the inclusion of two *VEGF* trials both investigating bevacizumab. Unfortunately, insufficient data was available regarding *HER2* status in most studies to permit subgroup analysis.

The review aimed to evaluate the overall effect of AAs in mOGC and to identify possible subgroups of greatest benefit to steer future research. As such, the review’s strengths lie in the comprehensive literature search, including hand-searching of relevant conference proceedings, as confirmed by the detection of several studies not found in prior literature searches (27); and the adherence to strict systematic review principles (via the PRISMA guidelines) including comprehensive quality assessment provide strong qualitative as well as quantitative review aspects to this article.

The above findings have several implications for clinical practice. They confirm that AAs have a place in the clinical management of mOGC to improve patient outcomes. There are currently insufficient data to support use of AAs in the first-line setting (based on the AVAGAST trial), although we note ongoing first-line studies of *VEGFR*-targeting agents such as apatinib (NCT02525237) and ramucirumab (NCT02314117). In contrast, there is sufficient evidence to support the use of AAs in later-line settings; for example, the use of ramucirumab combined with docetaxel in the second-line setting in patients suitably fit for chemotherapy, or single-agent ramucirumab if unfit (where available). For chemotherapy-refractory patients, the use of apatinib would be supported by current evidence. Whilst regorafenib has shown promise in a phase II trial, phase III data is awaited before determining its place in the clinical treatment paradigm. As immunotherapy agents are currently under exploration across multiple settings in gastric cancer, the landscape for drug therapy may change further depending on trial results, but the evidence for the use of AAs will remain. Potential future studies of interest may include the combination of AAs and immunotherapy.

We note that a meta-analysis on a similar subject was recently published by Qi et al[29]. We identified seven additional studies and more importantly rigorously evaluated the risk of bias in seven different domains, enabling accurate assessment of study quality before quantitative analysis. Our study is the first, to our knowledge, to confirm statistically greater efficacy of AAs in the refractory setting. This is likely due to the incorporation of positive trials for apatinib, regorafenib and ramucirumab with the updated literature search.

There are limitations in our study that should be acknowledged. The most significant limitation is the reliance on data in the public domain (including conference presentations), leading to the risk of publication bias. However, the funnel plot showed did not show significant asymmetry, supporting a low likelihood of publication bias. An individual patient data meta-analysis would increase the ability to detect real differences by subgroups. Some of the data used in our analyses have only been presented in abstract form thus far, and as full publications become available, full assessment of both risk of bias and outcomes will become feasible. In addition, given that the impact of *HER2* status has not been investigated (or at least published to date) for the identified trials, the above findings may be most relevant in the *HER2*-negative population. We chose not to perform subgroup analyses by gastrectomy status, given that the required subgroup results have not been published in the majority of identified trials.

Future studies could investigate the use of *VEGFR2*-targeted agents in the first-line setting, in a similar design to Yoon 2014 [18], reported in abstract form only thus far. Such agents have been proven in later-line settings and if they were equally efficacious in the first-line setting it may explain the apparent difference in efficacy noted above because of the different classes of AAs being investigated, rather than differences between tumour biology in different lines.

In summary, our review has identified that the addition of AAs to standard therapy improves outcomes in mOGC and that this benefit appears to be most certain with modern AAs (with Phase III data for ramucirumab and apatinib) when used as monotherapy in the chemo-refractory setting. Individual studies evaluating *VEGFR2* targeting agents have shown greatest benefit.

Our findings support ongoing research into the use of AAs in mOGC, particularly in identifying predictive biomarkers that may define their optimal place in the treatment paradigm of mOGC to maximise patient benefit from these agents.

## Supporting information

S1 Fig Risk of bias summary. (TIF)

S2 Fig Funnel plot—Overall Survival—all trials. (TIF)

S3 Fig Forest plot—Overall Grade 3—5 toxicity—all trials—random-effects modelling. (TIF)

S4 Fig Forest plot—Progression-free Survival—by line of therapy. (TIF)

S5 Fig Forest plot—Overall Grade 3—5 toxicity—by line of therapy—random-effects modelling. (TIF)

S6 Fig Forest plot—Progression-free Survival—by region. (TIF)

S7 Fig Forest plot—Overall Survival—by age. (TIF)

S8 Fig Forest plot—Overall Survival—by performance status (0 versus > = 1). (TIF)

S9 Fig Forest plot—Overall Survival—by histological subtype. (TIF)

S10 Fig Forest plot—Overall Survival—by number of metastatic sites (1—2 versus 3+). (TIF)

S11 Fig Forest plot—PFS—by mechanism of action. (TIF)

S12 Fig Forest plot—PFS—by mechanism of action—random effects modelling. (TIF)

S13 Fig Forest plot—Overall Survival—by location of primary. (TIF)

S1 Methods Search strategy for electronic databases. (DOCX)

S2 Methods PRISMA guidelines. (DOC)

S1 Table Quality of life data. (DOCX)
