# Supplementary figures and images for: The effect of anti-angiogenic agents on overall survival in metastatic oesophago-gastric cancer: A systematic review and meta-analysis
Source: PLoS One. 2017 Feb 21;12(2):e0172307. doi: 10.1371/journal.pone.0172307 (PMC5319652; doi:10.1371/journal.pone.0172307)

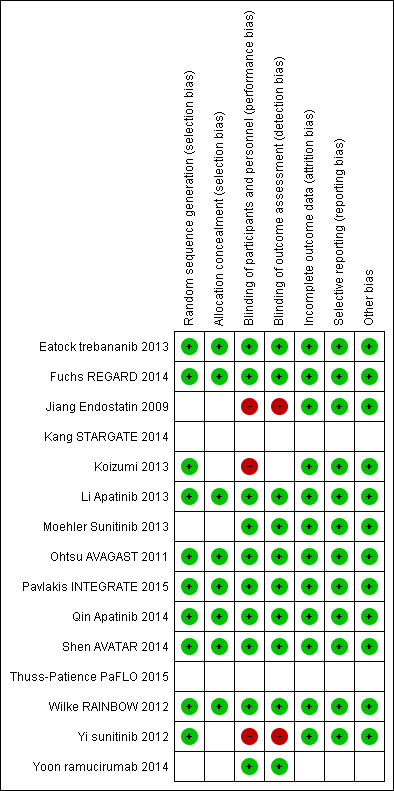

Supplement: S1 Fig — (TIF) [file pone.0172307.s001.tif]

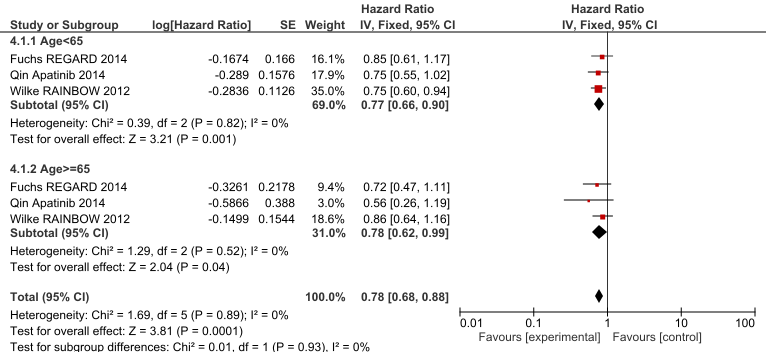

Supplement: S7 Fig — (TIF) [file pone.0172307.s007.tif]

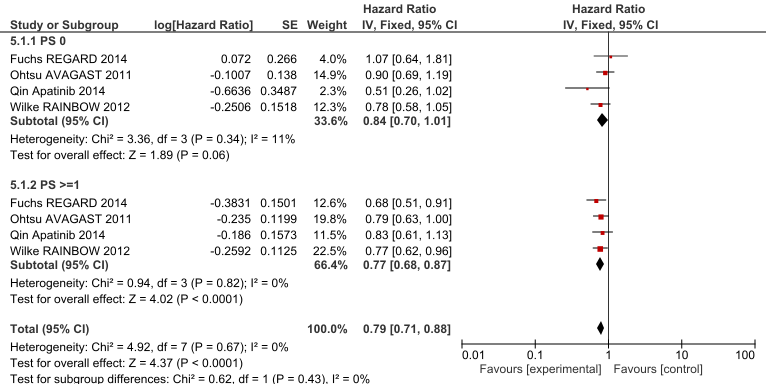

Supplement: S8 Fig — (TIF) [file pone.0172307.s008.tif]

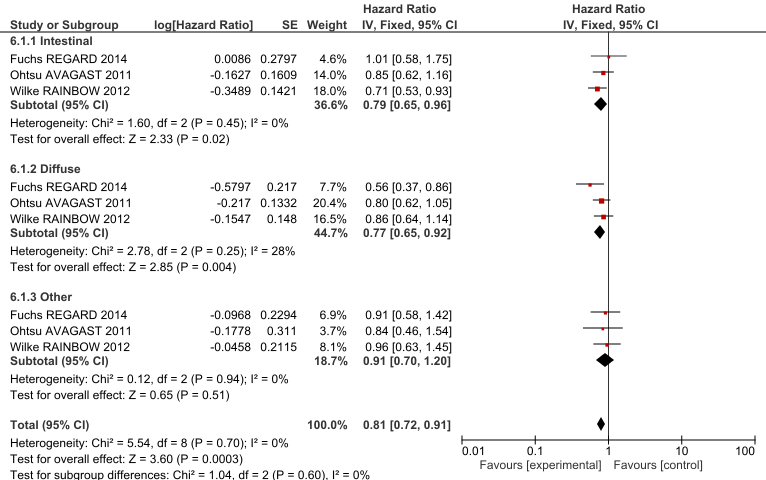

Supplement: S9 Fig — (TIF) [file pone.0172307.s009.tif]

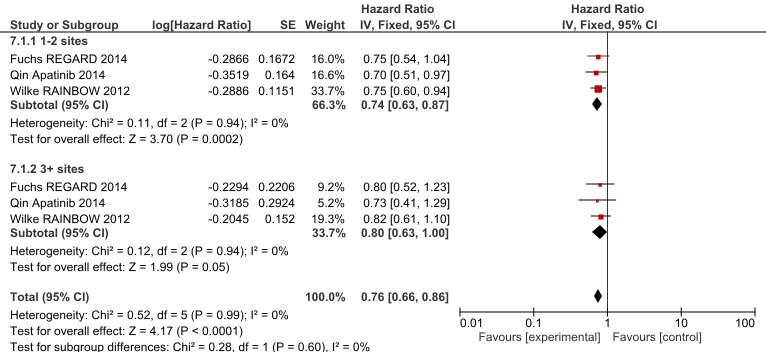

Supplement: S10 Fig — (TIF) [file pone.0172307.s010.tif]
