# Supplementary material for: The effect of anti-angiogenic agents on overall survival in metastatic oesophago-gastric cancer: A systematic review and meta-analysis
Source: PLoS One. 2017 Feb 21;12(2):e0172307. doi: 10.1371/journal.pone.0172307 (PMC5319652; doi:10.1371/journal.pone.0172307)
Supplement: S1 Methods — (DOCX) [file pone.0172307.s014.docx]

**Supp.Methods 1: Search strategy – Medline**

**Medline**

1. ((Stomach* or Esophagogastri* or Esophag*) adj3 (carcinom* or neoplas* or adenocarcinom* or cancer* or tumor* or tumour* or sarcom*)).mp.

2. exp Stomach Neoplasms/

3. exp Esophageal Neoplasms/

4. 1 or 2 or 3

5. (vascular endothelial growth factor or VEGF or VEGFR or FGFR or fibroblast growth factor or Bevacizumab or Aflibercept or Regorafenib or Brivanib or Pazopanib or Ramucirumab or Apatinib).mp.

6. exp Vascular Endothelial Growth Factors/

7. exp Receptors, Vascular Endothelial Growth Factor/

8. 5 or 6 or 7

9. exp Neoplasm Metastasis/

10. (metasta* or stage 4 or stage IV or advanced).mp.

11. 9 or 10

12. 4 and 8 and 11

13. randomized controlled trial.pt.

14. placebo.ab.

15. randomized.ab. or randomly.ab.

16. controlled clinical trial.pt. or clinical trial.sh. or trial.ti

17. 13 or 14 or (15 and 16)

18. humans.sh.

19. 17 and 18

20. 12 and 19
