# Supplementary material for: The effect of anti-angiogenic agents on overall survival in metastatic oesophago-gastric cancer: A systematic review and meta-analysis
Source: PLoS One. 2017 Feb 21;12(2):e0172307. doi: 10.1371/journal.pone.0172307 (PMC5319652; doi:10.1371/journal.pone.0172307)
Supplement: S1 Table — (DOCX) [file pone.0172307.s016.docx]

S1 Table. Quality of life

| Study title | Treatment | Control | Global QoL | Time to QoL deterioration | QoL subscales |
| --- | --- | --- | --- | --- | --- |
| Li 2013 | Apatinib | Placebo | EORTC QLQ-C30: No difference |  | Insomnia improved (p=0.0002) |
| REGARD | Ramucirumab | Placebo | EORTC QLQ-C30: No difference (p=0.23) | Time to ECOG>=2 favoured ramucirumab (HR 0.586, p=0.002) |  |
| Moehler | FOLFIRI +Sunitinib | FOLFIRI | “Performed quality of life outcomes were mostly in favor of Sunitinib.” |  |  |
| AVAGAST | CX +bevacizumab | CX | EORTC QLQ-C30/QLQ-STO22: No global difference |  | Slower deterioration in pain score with bevacizumab (p=0.0068) |
| RAINBOW | Paclitaxel +ramucirumab | Paclitaxel |  | EORTC QLQ-C30 – time to deterioration >10 points unchanged (HR 0.93, 95% CI 0.73-1.18) | Improved emotional functioning (HR 0.64, 95% CI 0.49-0.84) with ramucirumab but worsened diarrhoea (HR 1.33, 95% CI 1.01-1.76) |
| Jiang 2009 | XELOX +endostatin | XELOX | EORTC QLQ-C30 improved (p<0.05) |  |  |

Abbreviations: CX – Cisplatin + capecitabine, FOLFIRI – fluorouracil/folinic acid + irinotecan, XELOX – capecitabine+oxaliplatin
